# Transient Thermal Stress Problem of a Functionally Graded Magneto-Electro-Thermoelastic Hollow Sphere

**DOI:** 10.3390/ma4122136

**Published:** 2011-12-12

**Authors:** Yoshihiro Ootao, Masayuki Ishihara

**Affiliations:** Department of Mechanical Engineering, Graduate School of Engineering, Osaka Prefecture University, 1-1 Gakuen-cho, Naka-ku, Sakai 599-8531, Japan; E-Mail: ishihara@me.osakafu-u.ac.jp

**Keywords:** thermal stress, magneto-electro-thermoelastic material, functionally graded material, hollow sphere, transient state

## Abstract

This article is concerned with the theoretical analysis of the functionally graded magneto-electro-thermoelastic hollow sphere due to uniform surface heating. We analyze the transient thermoelastic problem for a functionally graded hollow sphere constructed of the spherical isotropic and linear magneto-electro-thermoelastic materials using a laminated composite mode as one of theoretical approximation in the spherically symmetric state. As an illustration, we carry out numerical calculations for a functionally graded hollow sphere constructed of piezoelectric and magnetostrictive materials and examine the behaviors in the transient state. The effects of the nonhomogeneity of material on the stresses, electric potential, and magnetic potential are investigated.

## 1. Introduction 

Functionally graded materials (FGMs) are new nonhomogeneous material systems that two or more different material ingredients changes continuously and gradually. The concept of FGMs is applicable to many industrial fields such as aerospace, nuclear energy, chemical plant, electronics and so on. On the other hand, it has recently been found that composites made of piezoelectric and magnetostrictive materials exhibit the magnetoelectric effect, which is not seen in piezoelectric or magnetostrictive materials [1]. These materials are known as multiferroic composites [2]. These composites exhibit a coupling among magnetic, electric, and elastic fields. In the past, various problems in magneto-electro-elastic media that exhibit anisotropic and linear coupling among the magnetic, electric, and elastic fields were analyzed. Examples for the plates and beams were analyzed in the papers [3,4,5]. Examples for the shell type structures were analyzed in the papers [6,7,8]. Examples of functionally graded magneto-electro-elastic media are as follows. Wang and Ding [9] treated spherically symmetric transient responses of a functionally graded magneto-electro-elastic hollow sphere. Ma and Lee [10] analyzed an in-plane problem in functionally graded magneto-electro-elastic bimaterials. Yu and Wu [11] analyzed the propagation of circumferential wave in magneto-electro-elastic functionally graded cylindrical curved plates. Wu and Lu [12] analyzed the 3D dynamics responses of functionally graded magneto-electro-elastic plates. Huang *et al*. [13] analyzed the static problem of an anisotropic functionally graded magneto-electro-elastic beams subjected to arbitrary loading. Lee and Ma [14] analyzed the two-dimensional problem of two bonded dissimilar half-planes for functionally graded magnetoelectroelastic materials subjected to generalized line forces and screw dislocations.

Examples of the thermal stress problems of electro-magneto-elastic media are as follows, Ganesan *et al*. [15] analyzed the response of a layered, multiphase magnetoelectroelastic cylinder subjected to an axisymmetric temperature distribution using finite element procedures. Kumaravel *et al*. [16] analyzed the response of a three-layered magnetoelectroelastic strip subjected to uniform temperature rise and non-uniform temperature distribution using finite element procedures. Hou *et al*. [17] obtained 2D fundamental solutions of a steady point heat source in infinite and semi-infinite orthotropic electro-magneto-thermo-elastic planes. With regard to transient thermal stress problems of electro-magneto-elastic media, Wang and Niraula [18] analyzed transient thermal fracture in transversely isotropic electro-magneto-elastic cylinders. The exact solution of a transient analysis of multilayered magneto-electro-thermoelastic strip subjected to nonuniform heat supply was obtained in the paper [19]. The exact solution of a transient analysis of multilayered magneto-electro-thermoelastic hollow cylinder subjected to uniform heat supply was obtained in the paper [20]. Though a several transient thermal stress problems of the functionally graded hollow spheres [21,22] using a laminated composite model were analyzed already, theses studies don’t consider a coupling among magnetic, electric, and thermoelastic fields. However, to the author’s knowledge, the transient thermal stress problem for a functionally graded magneto-electro-thermoelastic hollow spheres under unsteady heat supply considering a coupling among magnetic, electric, and thermoelastic fields has not been reported.

In the present article, we have analyzed the transient behavior of a functionally graded magneto-electro-thermoelastic hollow sphere due to uniform surface heating. We assumed that the magneto-electro-thermoelastic materials are polarized and magnetized in the radial direction. We analyze the transient thermal stress problem for a functionally graded hollow sphere constructed of the spherical isotropic and linear magneto-electro-thermoelastic materials using a laminated composite model as one of theoretical approximation. We carried out numerical calculations for a functionally graded hollow sphere composed of piezoelectric and magnetostrictive materials, and examined the effects of the nonhomogeneity of material on the stresses, electric potential, and magnetic potential.

## 2. Analysis

We consider a functionally graded hollow sphere constructed of the spherical isotropic and linear magneto-electro-thermoelastic materials. We analyze the transient thermal stress problem using a multilayered composite hollow sphere model with a number *N* of homogeneous layers. The hollow sphere’s inner and outer radii are designated *a* and *b*, respectively. *r_i_* is the outer radius of the *i*th layer. Throughout this article, the indices *i* (=1,2,…,*N*) are associated with the *i*th layer of a composite hollow sphere from the inner side.

### 2.1. Heat Conduction Problem

We assumed that the multilayered hollow sphere is initially at zero temperature and its inner and outer surfaces are suddenly heated by surrounding media having constant temperatures *T_a_* and *T_b_* with relative heat transfer coefficients *h_a_* and *h_b_*, respectively. Then, the temperature distribution is one-dimensional, and the transient heat conduction equation for the *i*th layer is written in the following form:

(1) $$\frac{\partial {\bar{T}}_{i}}{\partial \tau }={\bar{\kappa }}_{ri}(\frac{\partial^{2}{\bar{T}}_{i}}{\partial {\bar{r}}^{2}}+\frac{2}{\bar{r}}\frac{\partial {\bar{T}}_{i}}{\partial \bar{r}})\;;\;i=1,2,\cdots ,N$$

The initial and thermal boundary conditions in dimensionless form are

(2) $$\tau =0\;;\;{\bar{T}}_{i}=0\;;\;i=1,2,\cdots ,N$$

(3) $$\bar{r}=\bar{a}\;;\;\frac{\partial {\bar{T}}_{1}}{\partial \bar{r}}-H_{a}{\bar{T}}_{1}=-H_{a}{\bar{T}}_{a}$$

(4) $$\bar{r}=R_{i}\;;\;{\bar{T}}_{i}={\bar{T}}_{i+1}\;;\;i=1,2,\cdots ,N-1$$

(5) $$\bar{r}=R_{i};\;{\bar{\lambda }}_{ri}\frac{\partial {\bar{T}}_{i}}{\partial \bar{r}}={\bar{\lambda }}_{r,i+1}\frac{\partial {\bar{T}}_{i+1}}{\partial \bar{r}}\;;\;i=1,2,\cdots ,N-1$$

(6) $$\bar{r}=1;\;\frac{\partial {\bar{T}}_{N}}{\partial \bar{r}}+H_{b}{\bar{T}}_{N}=H_{b}{\bar{T}}_{b}$$

In Equations (1)–(6), we introduced the following dimensionless values:

(7) $$\begin{array}{c} ({\bar{T}}_{i},{\bar{T}}_{a},{\bar{T}}_{b})=(T_{i},T_{a},T_{b})/T_{0},(\bar{r},R_{i},\bar{a})=(r,r_{i},a)/b,\tau =\kappa_{0}t/b^{2}\\ {\bar{\kappa }}_{ri}\;=\kappa_{ri}/\kappa_{0},{\bar{\lambda }}_{ri}=\lambda_{ri}/\lambda_{0},\;(H_{a},H_{b})=(h_{a},h_{b})b \end{array}$$

where *T_i_* is the temperature change; *t* is time; *λ_ri_* is the thermal conductivity in the radial direction; *κ_ri_* is the thermal diffusivity in the radial direction; and *T_0_*, *λ_0_* and *κ_0_* are typical values of temperature, thermal conductivity, and thermal diffusivity, respectively. To solve the fundamental equation (1), we introduced the Laplace transformation with respect to the variable *τ* as follows;

(8) $${\bar{T}}_{i}^{*}(\bar{r},p)=\int_{0}^{\infty }{\bar{T}}_{i}(\bar{r},\tau )e^{-p\tau }d\tau$$

Performing the Laplace transformation on Equation (1) under the condition of Equation (2) gives

(9) $$\frac{d^{2}{\bar{T}}_{i}^{*}}{d{\bar{r}}^{2}}+\frac{2}{\bar{r}}\frac{d{\bar{T}}_{i}^{*}}{d\bar{r}}+\mu^{2}\beta_{i}^{2}{\bar{T}}_{i}^{*}=0$$

where

(10) $$\mu^{2}=-p,\beta_{i}=\frac{1}{\sqrt{{\bar{\kappa }}_{ri}}}$$

The general solution of Equation (9) is

(11) $${\bar{T}}_{i}^{*}=A_{i}j_{0}(\beta_{i}\mu \bar{r})+B_{i}y_{0}(\beta_{i}\mu \bar{r})$$

where *j*_0_() and *y*_0_() are zeroth-order spherical Bessel functions of the first and second kind, respectively. Furthermore, *A_i_* and *B_i_* are unknown constants determined from the boundary conditions. Substituting Equation (11) into the boundary conditions in the transformed domain from Equations (3)–(6), these equations are represented in matrix form as follows:

(12) $$\left[a_{kl}\right]\left\{\begin{array}{c} A_{1}\\ B_{1}\\ \vdots \\ A_{N}\\ B_{N} \end{array}\right\}=\frac{1}{p}\left\{c_{k}\right\}$$

Making use of Cramer’s formula, the constants *A_i_* and *B_i_* can be determined from Equation (12). Then the temperature solution in the transformed domain is

(13) $${\bar{T}}_{i}^{*}=\frac{1}{p\Delta }[{\bar{A}}_{i}j_{0}(\beta_{i}\mu \bar{r})+{\bar{B}}_{i}y_{0}(\beta_{i}\mu \bar{r})]$$

where Δ is the determinant of 2*N* × 2*N* matrix [*a_kl_*], and the coefficients ${\bar{A}}_{i}$ and ${\bar{B}}_{i}$ are defined as determinants of a matrix similar to the coefficient matrix [*a_kl_*], in which the (2*i* − 1)th column or 2*i*th column is replaced with the constant vector {*c_k_*}, respectively. Using the residue theorem, we can accomplish the inverse Laplace transformation on Equation (13). Because the single-valued poles of Equation (13) correspond to *p* = 0 and the roots of Δ = 0, in which the residue for *p* = 0 gives a solution for the steady state. Accomplishing the inverse Laplace transformation of Equation (13), the solution of Equation (1) is written as follows:

(14) $${\bar{T}}_{i}=\frac{1}{F}({{\bar{A}}^{\prime }}_{i}\;+\frac{{{\bar{B}}^{\prime }}_{i}}{\bar{r}}\;)+\sum_{j=1}^{\infty }\frac{2\exp(-\mu_{j}^{2}\tau )}{\mu_{j}\Delta^{\prime }(\mu_{j})}[{\bar{A}}_{i}j_{0}(\beta_{i}\mu_{j}\bar{r})+{\bar{B}}_{i}y_{0}(\beta_{i}\mu_{j}\bar{r})];i=1,2,\cdots ,N$$

where *F* ie the determinants of 2*N* × 2*N* matrix [*e_kl_*], and the coefficients ${{\bar{A}}^{\prime }}_{i}$ and ${{\bar{B}}^{\prime }}_{i}$ are defined as determinants of a matrix similar to the coefficient matrix [*e_kl_*], in which the (2*i* − 1)th column or 2*i*th column is replaced with the constant vector {*c_k_*}, respectively. The nonzero elements of the coefficient matrices [*a_kl_*] and [*e_kl_*] and the constant vector {*c_k_*} are given from the Equations (3)–(6). In Equation (14), $\Delta^{\prime }(\mu_{j})$ is

(15) $$\Delta^{\prime }(\mu_{j})={\frac{d\Delta }{d\mu }|}_{\mu =\mu_{j}}$$

and *μ_j_* is the *j*th positive root of the following transcendental equation

(16) Δ(μ)=0

### 2.2. Thermoelastic Problem

We developed the analysis of a multilayered magneto-electro-thermoelastic hollow sphere as a spherically symmetric state. The displacement-strain relations are expressed in dimensionless form as follows:

(17) $${\bar{\varepsilon }}_{rri}={\bar{u}}_{ri}{,}_{\bar{r}},{\bar{\varepsilon }}_{\theta \theta i}={\bar{\varepsilon }}_{\varphi \varphi i}=\frac{{\bar{u}}_{ri}}{\bar{r}},{\bar{\gamma }}_{r\theta i}={\bar{\gamma }}_{r\varphi i}={\bar{\gamma }}_{\theta \varphi i}=0$$

where the comma denotes partial differentiation with respect to the variable that follows. For the spherical isotropic and linear magneto-electro-thermoelastic material, the constitutive relations are expressed in dimensionless form as follows:

(18) $$\begin{array}{c} {\bar{\sigma }}_{rri}={\bar{C}}_{11i}{\bar{\varepsilon }}_{rri}+2{\bar{C}}_{12i}{\bar{\varepsilon }}_{\theta \theta i}-{\bar{\beta }}_{ri}{\bar{T}}_{i}-{\bar{e}}_{1i}{\bar{E}}_{ri}-{\bar{q}}_{1i}{\bar{H}}_{ri}\\ {\bar{\sigma }}_{\theta \theta i}={\bar{\sigma }}_{\varphi \varphi i}={\bar{C}}_{12i}{\bar{\varepsilon }}_{rri}+({\bar{C}}_{22i}+{\bar{C}}_{23i}){\bar{\varepsilon }}_{\theta \theta i}-{\bar{\beta }}_{\theta i}{\bar{T}}_{i}-{\bar{e}}_{2i}{\bar{E}}_{ri}-{\bar{q}}_{2i}{\bar{H}}_{ri} \end{array}$$

where

(19) $${\bar{\beta }}_{ri}={\bar{C}}_{11i}{\bar{\alpha }}_{ri}+2{\bar{C}}_{12i}{\bar{\alpha }}_{\theta i},{\bar{\beta }}_{ri}={\bar{C}}_{11i}{\bar{\alpha }}_{ri}+2{\bar{C}}_{12i}{\bar{\alpha }}_{\theta i}$$

The constitutive equations for the electric and the magnetic fields in dimensionless form are given as

(20) $${\bar{D}}_{ri}={\bar{e}}_{1i}{\bar{\varepsilon }}_{rri}+2{\bar{e}}_{2i}{\bar{\varepsilon }}_{\theta \theta i}+{\bar{\eta }}_{1i}{\bar{E}}_{ri}+{\bar{d}}_{1i}{\bar{H}}_{ri}+{\bar{p}}_{1i}{\bar{T}}_{i}$$

(21) $${\bar{B}}_{ri}={\bar{q}}_{1i}{\bar{\varepsilon }}_{rri}+2{\bar{q}}_{2i}{\bar{\varepsilon }}_{\theta \theta i}+{\bar{d}}_{1i}{\bar{E}}_{ri}+{\bar{\mu }}_{1i}{\bar{H}}_{ri}+{\bar{m}}_{1i}{\bar{T}}_{i}$$

The relation between the electric field intensity and the electric potential $\varphi_{i}$ in dimensionless form is defined as

(22) $${\bar{E}}_{ri}=-{\bar{\varphi }}_{i}{,}_{\bar{r}}$$

The relation between the magnetic field intensity and the magnetic potential $\psi_{i}$ in dimensionless form is defined as

(23) $${\bar{H}}_{ri}=-{\bar{\psi }}_{i}{,}_{\bar{r}}$$

The equilibrium equation is expressed in dimensionless form as follows:

(24) $${\bar{\sigma }}_{rri}{,}_{\bar{r}}+\frac{2}{\bar{r}}({\bar{\sigma }}_{rri}-{\bar{\sigma }}_{\theta \theta \;i})=0$$

If the electric charge density is absent, the equations of electrostatics and magnetostatics are expressed in dimensionless form as follows:

(25) $${\bar{D}}_{ri}{,}_{\bar{r}}+\frac{2{\bar{D}}_{ri}}{\bar{r}}=0$$

(26) $${\bar{B}}_{ri}{,}_{\bar{r}}+\frac{2{\bar{B}}_{ri}}{\bar{r}}=0$$

In Equations (17)–(26), the following dimensionless values are introduced:

(27) $$\begin{array}{c} {\bar{\sigma }}_{kli}=\frac{\sigma_{kli}}{\alpha_{0}Y_{0}T_{0}},({\bar{\varepsilon }}_{kli},{\bar{\gamma }}_{kli})=\frac{\left(\varepsilon_{kli},\gamma_{kli}\right)}{\alpha_{0}T_{0}},{\bar{u}}_{ri}=\frac{u_{ri}}{\alpha_{0}T_{0}b},{\bar{\alpha }}_{ki}=\frac{\alpha_{ki}}{\alpha_{0}},{\bar{C}}_{kli}=\frac{C_{kli}}{Y_{0}}\\ {\bar{D}}_{ri}=\frac{D_{ri}}{\alpha_{0}Y_{0}T_{0}\left|d_{0}\right|},{\bar{B}}_{ri}=\frac{B_{ri}\left|d_{0}\right|\kappa_{0}}{b\alpha_{0}T_{0}},{\bar{\varphi }}_{i}=\frac{\varphi_{i}\left|d_{0}\right|}{\alpha_{0}T_{0}b},{\bar{\psi }}_{i}=\frac{\psi_{i}}{\left|d_{0}\right|\kappa_{0}\alpha_{0}Y_{0}T_{0}},\\ {\bar{e}}_{ki}=\frac{e_{ki}}{Y_{0}\left|d_{0}\right|},{\bar{\eta }}_{1i}=\frac{\eta_{1i}}{Y_{0}{\left|d_{0}\right|}^{2}},{\bar{q}}_{ki}=\frac{q_{ki}\kappa_{0}\left|d_{0}\right|}{b},{\bar{\mu }}_{1i}=\frac{\mu_{1i}\kappa_{0}^{2}{\left|d_{0}\right|}^{2}Y_{0}}{b^{2}},\\ {\bar{d}}_{1i}=\frac{\kappa_{0}d_{1i}}{b},{\bar{p}}_{1i}=\frac{p_{1i}}{\alpha_{0}Y_{0}\left|d_{0}\right|},{\bar{m}}_{1i}=\frac{m_{1i}\kappa_{0}\left|d_{0}\right|}{b\alpha_{0}},{\bar{E}}_{ri}=\frac{E_{ri}\left|d_{0}\right|}{\alpha_{0}T_{0}},{\bar{H}}_{ri}=\frac{H_{ri}b}{\left|d_{0}\right|\kappa_{0}\alpha_{0}Y_{0}T_{0}} \end{array}$$

where $\sigma_{kli}$ are the stress components; ($\varepsilon_{kli}$, $\gamma_{kli}$) are the strain components; *u_ri_* is the displacement in the *r* direction; *α_ri_* are the coefficients of linear thermal expansion; *C_kli_* are the elastic stiffness constants; *D_ri_* is the electric displacement in the *r* direction; *B_ri_* is the magnetic flux density in the *r* direction; *e_ki_* are the piezoelectric coefficients; *η*_1*i*_ is the dielectric constant; *P*_1*i*_ is the pyroelectric constant; *q_ki_* are the piezomagnetic coefficients; *μ*_1*i*_ is the magnetic permeability coefficient; *d*_1*i*_ is the magnetoelectric coefficient; *m*_1*i*_ is the pyromagnetic constant; and *α*_0_, *Y*_0_ and *d*_0_ are typical values of the coefficient of linear thermal expansion, Young’s modulus, and piezoelectric modulus, respectively.

Substituting Equations (17), (22), and (23) into Equations (18), (20), and (21) and later into Equations (24)–(26), the governing equations of the displacement *u_ri_*, electric potential *φ_i_*, and magnetic potential *ψ_i_* in the dimensionless form are written as

(28) $$\begin{array}{c} {\bar{C}}_{11i}{\bar{u}}_{ri}{,}_{\bar{r}\bar{r}}+2{\bar{C}}_{11i}{\bar{u}}_{ri}{,}_{\bar{r}}{\bar{r}}^{-1}+2({\bar{C}}_{12i}-{\bar{C}}_{22i}-{\bar{C}}_{23i}){\bar{u}}_{ri}{\bar{r}}^{-2}+{\bar{e}}_{1i}{\bar{\varphi }}_{i}{,}_{\bar{r}\hat{r}}+2({\bar{e}}_{1i}-{\bar{e}}_{2i}){\bar{\varphi }}_{i}{,}_{\bar{r}}{\bar{r}}^{-1}\\ +{\bar{q}}_{1i}{\bar{\psi }}_{i}{,}_{\bar{r}\bar{r}}+2({\bar{q}}_{1i}-{\bar{q}}_{2i}){\bar{\psi }}_{i}{,}_{\bar{r}}{\bar{r}}^{-1}=2({\bar{\beta }}_{ri}-{\bar{\beta }}_{\theta i}){\bar{T}}_{i}{\bar{r}}^{-1}+{\bar{\beta }}_{ri}{\bar{T}}_{i}{,}_{\bar{r}} \end{array}$$

(29) $$\begin{array}{c} {\bar{e}}_{1i}{\bar{u}}_{ri}{,}_{\bar{r}\bar{r}}+2({\bar{e}}_{1i}+{\bar{e}}_{2i}){\bar{u}}_{ri}{,}_{\bar{r}}{\bar{r}}^{-1}+2{\bar{e}}_{2i}{\bar{u}}_{ri}{\bar{r}}^{-2}-{\bar{\eta }}_{1i}{\bar{\varphi }}_{i}{,}_{\bar{r}\bar{r}}-2{\bar{\eta }}_{1i}{\bar{\varphi }}_{i}{,}_{\bar{r}}{\bar{r}}^{-1}\\ -{\bar{d}}_{1i}{\bar{\psi }}_{i}{,}_{\bar{r}\hat{r}}-2{\bar{d}}_{1i}{\bar{\psi }}_{i}{,}_{\bar{r}}{\bar{r}}^{-1}=-{\bar{p}}_{1i}({\bar{T}}_{i}{,}_{\bar{r}}+2{\bar{T}}_{i}{\bar{r}}^{-1}) \end{array}$$

(30) $$\begin{array}{c} {\bar{q}}_{1i}{\bar{u}}_{ri}{,}_{\bar{r}\bar{r}}+2({\bar{q}}_{1i}+{\bar{q}}_{2i}){\bar{u}}_{ri}{,}_{\bar{r}}{\bar{r}}^{-1}+2{\bar{q}}_{2i}{\bar{u}}_{ri}{\bar{r}}^{-2}-{\bar{d}}_{1i}{\bar{\varphi }}_{i}{,}_{\bar{r}\bar{r}}-2{\bar{d}}_{1i}{\bar{\varphi }}_{i}{,}_{\bar{r}}{\bar{r}}^{-1}\\ -{\bar{\mu }}_{1i}{\bar{\psi }}_{i}{,}_{\bar{r}\bar{r}}-2{\bar{\mu }}_{1i}{\bar{\psi }}_{i}{,}_{\bar{r}}{\bar{r}}^{-1}-{\bar{\mu }}_{1i}{\bar{\psi }}_{i}{,}_{\bar{r}\bar{r}}-2{\bar{\mu }}_{1i}{\bar{\psi }}_{i}{,}_{\bar{r}}{\bar{r}}^{-1} \end{array}$$

If the inner and outer surfaces of the multilayered magneto-electro-thermoelastic hollow sphere are traction free, and the interfaces of each adjoining layer are perfectly bonded, then the boundary conditions of inner and outer surfaces and the conditions of continuity at the interfaces can be represented as follows:

(31) $$\begin{array}{c} \bar{r}=\bar{a};{\bar{\sigma }}_{rr1}=0\\ \bar{r}=R_{i};{\bar{\sigma }}_{rri}={\bar{\sigma }}_{rr,i+1},{\bar{u}}_{ri}={\bar{u}}_{r,i+1};\;i=1,2,\cdots ,N-1\\ \bar{r}=1;{\bar{\sigma }}_{rrN}=0 \end{array}$$

The boundary conditions in the radial direction for the electric and magnetic fields are expressed as
(32)
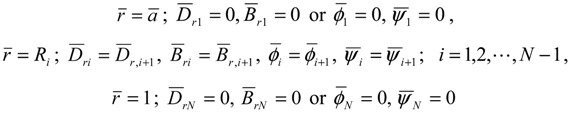


The solutions of Equations (28)–(30) are assumed in the following form:

(33) $${\bar{u}}_{ri}={\bar{u}}_{rci}+{\bar{u}}_{rpi},{\bar{\varphi }}_{i}={\bar{\varphi }}_{ci}+{\bar{\varphi }}_{pi},{\bar{\psi }}_{i}={\bar{\psi }}_{ci}+{\bar{\psi }}_{pi}$$

In Equation (33), the first term on the right-hand side gives the homogeneous solution and the second term gives the particular solution. We now consider the homogeneous solution, and introduce the following equation:

(34) $$\bar{r}=\exp(s)$$

Changing a variable with the use of Equation (34), the homogeneous expression of Equations (28)–(30) are

(35) $$[{\bar{D}}^{2}+\bar{D}-\alpha_{i}]{\bar{u}}_{rci}+[{\bar{D}}^{2}+\bar{D}-\beta_{ei}\bar{D}]{\bar{\Phi }}_{ci}+[{\bar{D}}^{2}+\bar{D}-\beta_{qi}\bar{D}]{\bar{\Psi }}_{ci}=0$$

(36) $$[{\bar{D}}^{2}+\bar{D}+\beta_{ei}(\bar{D}+1)]{\bar{u}}_{rci}-\gamma_{i}({\bar{D}}^{2}+\bar{D}){\bar{\Phi }}_{ci}-\beta_{di}({\bar{D}}^{2}+\bar{D}){\bar{\Psi }}_{ci}=0$$

(37) $$[{\bar{D}}^{2}+\bar{D}+\beta_{qi}(\bar{D}+1)]{\bar{u}}_{rci}-\beta_{di}({\bar{D}}^{2}+\bar{D}){\bar{\Phi }}_{ci}-\delta_{i}({\bar{D}}^{2}+\bar{D}){\bar{\Psi }}_{ci}=0$$

where

(38) $$\bar{D}=\frac{d}{ds}$$

(39) $$\begin{array}{c} {\bar{\Phi }}_{ci}=\frac{{\bar{e}}_{1i}}{{\bar{C}}_{11i}}{\bar{\varphi }}_{ci},{\bar{\Psi }}_{ci}=\frac{{\bar{q}}_{1i}}{{\bar{C}}_{11i}}{\bar{\psi }}_{ci},\alpha_{i}=\frac{2({\bar{C}}_{22i}+{\bar{C}}_{23i}-{\bar{C}}_{12i})}{{\bar{C}}_{11i}},\\ \beta_{ei}=\frac{2{\bar{e}}_{2i}}{{\bar{e}}_{1i}},\beta_{qi}=\frac{2{\bar{q}}_{2i}}{{\bar{q}}_{1i}},\beta_{di}=\frac{{\bar{C}}_{11i}{\bar{d}}_{1i}}{{\bar{e}}_{1i}{\bar{q}}_{1i}},{\bar{\gamma }}_{i}=\frac{{\bar{C}}_{11i}{\bar{\eta }}_{1i}}{{\bar{e}}_{1i}^{2}},\delta_{i}=\frac{{\bar{C}}_{11i}{\bar{\mu }}_{1i}}{{\bar{q}}_{1i}^{2}} \end{array}$$

By eliminating ${\bar{\Phi }}_{ci}$ and ${\bar{\Psi }}_{ci}$ between Eqs. (35)-(37), we can obtain an ordinary differential equation about ${\bar{u}}_{rci}$:

(40) $$b_{3i}\bar{D}{\left(\bar{D}+1\right)}^{2}{\bar{u}}_{rci}+b_{1i}(\bar{D}+1){\bar{u}}_{rci}=0$$

The solution of Equation (40) can be expressed as follows when $1-4b_{1i}/b_{3i}>0$.

(41) $${\bar{u}}_{rci}=C_{1i}{\bar{r}}^{-1}+C_{2i}{\bar{r}}^{\lambda_{i2}}+C_{3i}{\bar{r}}^{\lambda_{i3}}$$

From Equations (36), (37) and (41), we can obtain an ordinary differential equation about ${\bar{\Psi }}_{ci}$:

(42) $$\bar{D}{\bar{\Psi }}_{ci}+{\bar{\Psi }}_{ci}=a_{3i}C_{2i}e^{\lambda_{i2}s}+a_{4i}C_{3i}e^{\lambda_{i3}s}+C_{4i}$$

Using Equation (39), the solution of Equation (42) is

(43) $${\bar{\psi }}_{ci}=\frac{{\bar{C}}_{11i}}{{\bar{q}}_{1i}}(C_{4i}+C_{5i}{\bar{r}}^{-1}+g_{3i}C_{2i}{\bar{r}}^{\lambda_{i2}}+g_{4i}C_{3i}{\bar{r}}^{\lambda_{i3}})$$

From Equations (36), (41) and (43), we can obtain an ordinary differential equation about ${\bar{\Phi }}_{ci}$:

(44) $$\bar{D}{\bar{\Phi }}_{ci}+{\bar{\Phi }}_{ci}=a_{1i}C_{2i}e^{\lambda_{i2}s}+a_{2i}C_{3i}e^{\lambda_{i3}s}+C_{6i}$$

Using Equation (39), the solution of Equation (44) is

(45) $${\bar{\varphi }}_{ci}=\frac{{\bar{C}}_{11i}}{{\bar{e}}_{1i}}(C_{6i}+C_{7i}{\bar{r}}^{-1}+g_{1i}C_{2i}{\bar{r}}^{\lambda_{i2}}+g_{2i}C_{3i}{\bar{r}}^{\lambda_{i3}})$$

In Equations (41)–(45)

(46) $$\begin{array}{c} \lambda_{i2},\lambda_{i3}=\frac{-1\pm \sqrt{1-4b_{1i}/b_{3i}}}{2},\\ b_{1i}=-[\alpha_{i}\gamma_{i}+\beta_{ei}^{2}-\beta_{ei}-\frac{\beta_{qi}\gamma_{i}-\beta_{ei}\beta_{di}}{\delta_{i}\gamma_{i}-\beta_{di}^{2}}(\gamma_{i}-\beta_{di}-\beta_{qi}\gamma_{i}+\beta_{ei}\beta_{di})]\\ b_{3i}=1+\gamma_{i}+\frac{{\left(\gamma_{i}-\beta_{di}\right)}^{2}}{\delta_{i}\gamma_{i}-\beta_{di}^{2}}\\ g_{1i}=\frac{a_{1i}}{\lambda_{i2}+1},g_{2i}=\frac{a_{2i}}{\lambda_{i3}+1},g_{3i}=\frac{a_{3i}}{\lambda_{i2}+1},g_{4i}=\frac{a_{4i}}{\lambda_{i3}+1},\\ a_{1i}=\frac{1}{\gamma_{i}\lambda_{i2}}[\lambda_{i2}^{2}+\lambda_{i2}+\beta_{ei}(\lambda_{i2}+1)-\beta_{di}\lambda_{i2}a_{3i}]\\ a_{2i}=\frac{1}{\gamma_{i}\lambda_{i3}}[\lambda_{i3}^{2}+\lambda_{i3}+\beta_{ei}(\lambda_{i3}+1)-\beta_{di}\lambda_{i3}a_{4i}]\\ a_{3i}=\frac{\lambda_{i2}+1}{\lambda_{i2}(\delta_{i}\gamma_{i}-\beta_{di}^{2})}[(\gamma_{i}-\beta_{di})\lambda_{i2}+\beta_{qi}\gamma_{i}-\beta_{ei}\beta_{di}]\\ a_{4i}=\frac{\lambda_{i3}+1}{\lambda_{i3}(\delta_{i}\gamma_{i}-\beta_{di}^{2})}[(\gamma_{i}-\beta_{di})\lambda_{i3}+\beta_{qi}\gamma_{i}-\beta_{ei}\beta_{di}] \end{array}$$

In Equations (41), (43) and (45), *C_ki_* (k=1,2,⋯,7) are unknown constants. We have the following relation.

(47) $$-\alpha_{i}C_{1i}+\beta_{ei}C_{7i}+\beta_{qi}C_{5i}=0$$

The homogeneous solutions when $1-4b_{1i}/b_{3i}\leq 0$ are omitted here for brevity.

It is difficult to obtain the particular solutions using the temperature solution of Equation (14). In order to obtain the particular solutions, series expansions of Bessel functions given in Equation (14) are used. Equation (14) can be written in the following way:

(48) $${\bar{T}}_{i}(\bar{r},\tau )=\sum_{n=0}^{\infty }\left[a_{in}(\tau ){\bar{r}}^{2n}+b_{in}(\tau ){\bar{r}}^{2n-1}\right]$$

where

(49) $$\begin{array}{c} a_{in}(\tau )=\frac{{{\bar{A}}^{\prime }}_{i}}{F}\delta_{0n}+\sum_{j=1}^{\infty }{\bar{A}}_{i}\frac{2\exp(-\mu_{j}^{2}\tau )}{\mu_{j}\Delta^{\prime }(\mu_{j})}\cdot \frac{{\left(-1\right)}^{n}{\left(\beta_{i}\mu_{j}\right)}^{2n}}{(2n+1)!},\\ b_{in}(\tau )=\frac{{{\bar{B}}^{\prime }}_{i}}{F}\delta_{0n}+\sum_{j=1}^{\infty }{\bar{B}}_{i}\frac{2\exp(-\mu_{j}^{2}\tau )}{\mu_{j}\Delta^{\prime }(\mu_{j})}\cdot \frac{{\left(-1\right)}^{n+1}{\left(\beta_{i}\mu_{j}\right)}^{2n-1}}{(2n)!} \end{array}$$

Here, $\delta_{0n}$ is the Kronecker delta. The particular solutions ${\bar{u}}_{rpi}$, ${\bar{\varphi }}_{ip}$, and ${\bar{\psi }}_{pi}$ are obtained as the function system like Equation (48). Then, the stress components, electric displacement, and magnetic flux density can be evaluated from Equations (41), (43) and (45). Details of the solutions are omitted from here for brevity. The unknown constants in the homogeneous solutions are determined so as to satisfy the boundary conditions in (31) and (32).

## 3. Numerical Results

To illustrate the foregoing analysis, we consider the functionally graded hollow sphere composed of piezoelectric and magnetostrictive materials. The piezoelectric material is made up of BaTiO_3_, and the magnetostrictive material is made up of CoFe_2_O_4_. Numerical parameters of heat conduction and shape are presented as follows:

(50) $$\begin{array}{c} H_{a}=H_{b}=1.0,{\bar{T}}_{a}=0,{\bar{T}}_{b}=1,N=2,\;10,\\ \bar{a}=0.7,R_{i}-R_{i-1}=(1-\bar{a})/N,b=0.01m \end{array}$$

The first layer is pure piezoelectric material and the *N*th layer is pure magnetostrictive material. It is assumed that the volume fractions of the piezoelectric phase *V_p_* and the magnetostrictive phase *V_m_* for other layers are given by the relations

(51) $$\begin{array}{c} V_{p}={\left[1-\left(\frac{\bar{r}-\bar{a}}{1-\bar{a}}\right)\right]}^{1/M}\text{ if }0\leq M\leq 1,\\ V_{p}=1-{\left(\frac{\bar{r}-\bar{a}}{1-\bar{a}}\right)}^{M}\text{ if }M\geq 1,V_{m}=1-V_{p} \end{array}$$

The value of *V_p_* in *i*th layer is obtained by calculating the value of *V_p_* in Equation (34) at the centre point of each layer defined by $\bar{r}=(R_{i-1}+R_{i})/2$. To estimate the material properties of FGM, we apply the simplest linear law of mixture. The material constants considered for BaTiO_3_ and CoFe_2_O_4_ are shown in the paper [20]. The typical values of material parameters such as *κ_0_*, *λ_0_*, *α_0_*, *Y_0_*, and *d_0_*, used to normalize the numerical data, based on those of BaTiO_3_ are as follows:

(52) $$\kappa_{0}=\kappa_{r},\lambda_{0}=\lambda_{r},\alpha_{0}=\alpha_{\theta },Y_{0}=116\;GPa,d_{0}=-78\times 10^{-12}\;C/N$$

In the numerical calculations, the boundary conditions at the surfaces for the electric and magnetic fields are expressed as

(53) $$\begin{array}{c} \bar{r}=\bar{a};{\bar{D}}_{r1}=0,\;{\bar{B}}_{r1}=0\\ \bar{r}=1;{\bar{\varphi }}_{N}=0,\;{\bar{\psi }}_{N}=0 \end{array}$$

**Figure 1:**
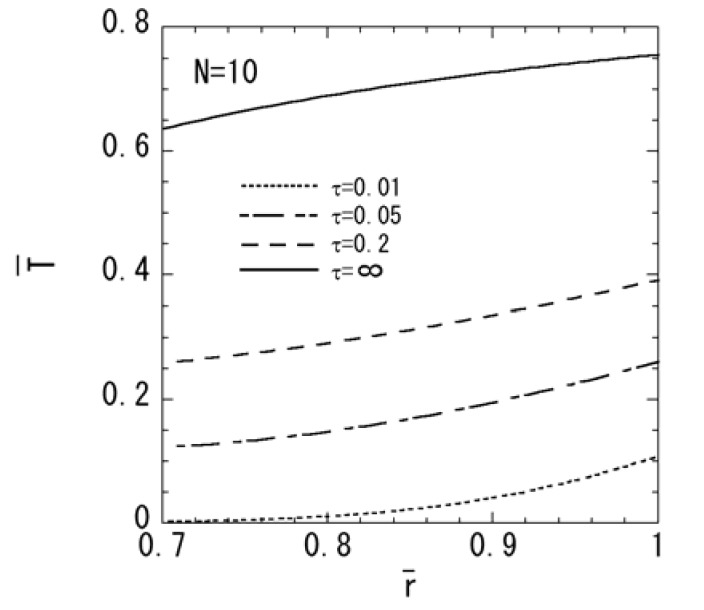
Variation of the temperature change (*M* = 1, *N* = 10).

**Figure 2:**
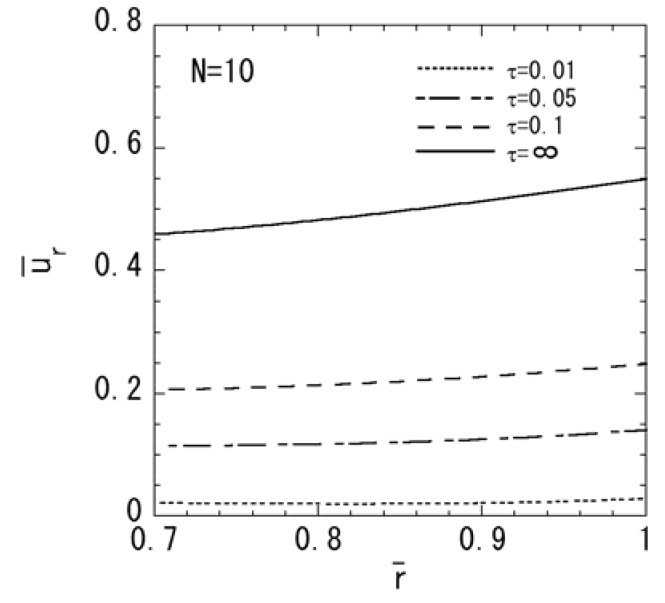
Variation of the displacement ${\bar{u}}_{r}$ (*M* = 1, *N* = 10)

**Figure 3:**
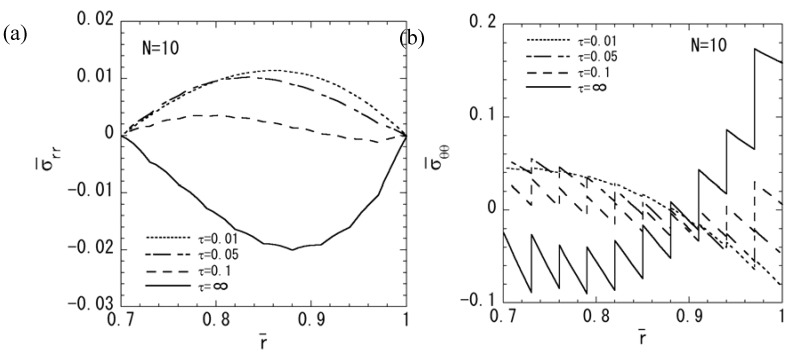
Variation of the thermal stresses (*M* = 1, *N* = 10): (**a**) normal stress ${\bar{\sigma }}_{rr}$; (**b**) normal stress ${\bar{\sigma }}_{\theta \theta }$.

**Figure 4:**
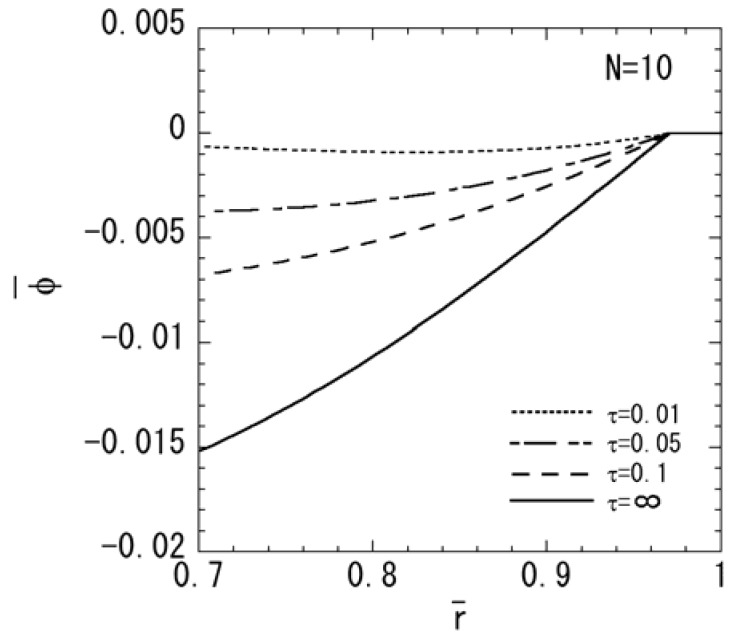
Variation of the electric potential (*M* = 1, *N* = 10).

**Figure 5:**
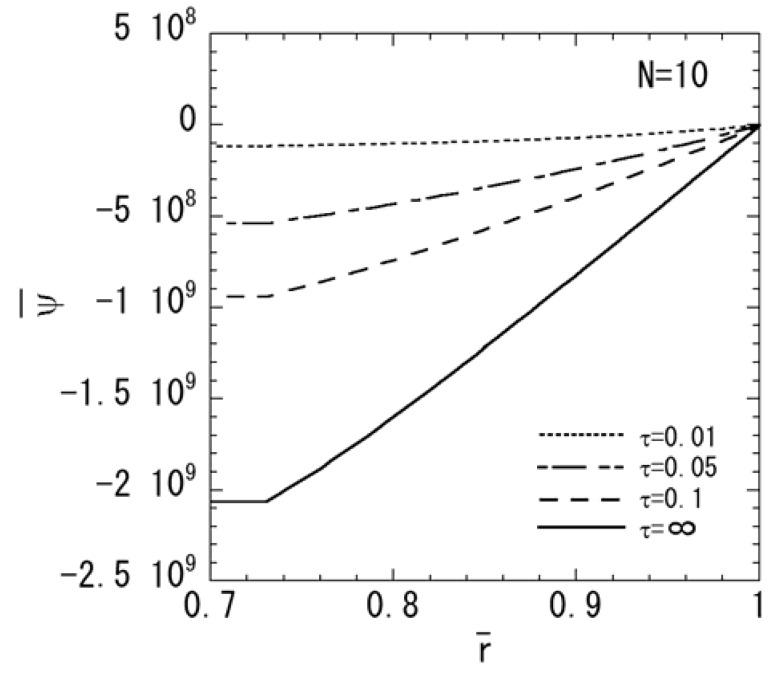
Variation of the magnetic potential (*M* = 1, *N* = 10).

Figure 1, Figure 2, Figure 3, Figure 4 and Figure 5 show the numerical results for *M* = 1 and *N* = 10. The variations of temperature change and displacement ${\bar{u}}_{r}$, along the radial direction are shown in Figure 1 and Figure 2, respectively. From Figure 1 and Figure 2, it is clear that the temperature and displacement increase with time and have the largest values in the steady state. The variations of normal stresses ${\bar{\sigma }}_{rr}$ and ${\bar{\sigma }}_{\theta \theta }$ along the radial direction are shown in Figure 3a and Figure 3b, respectively. Figure 3a reveals that the maximum tensile stress of ${\bar{\sigma }}_{rr}$ occurs in the transient state and the maximum compressive stress of ${\bar{\sigma }}_{rr}$ occurs in the steady state. From Figure 3b, it is clear that the maximum tensile stress occurs near the outer surface. The variations of electric potential $\bar{\phi }$ and magnetic potential $\bar{\psi }$ along the radial direction are shown in Figure 4 and Figure 5, respectively. Figure 4 reveals that the absolute value of the electric potential increases with time, and attains its maximum value in the steady state. The electric potential is almost zero in the tenth layer, i.e. the pure magnetostrictive layer. From Figure 5, it is clear that the absolute value of the magnetic potential increases with time and attains its maximum value in the steady state. The magnetic potential is almost constant in the first layer, i.e. the pure piezoelectric layer.

**Figure 6:**
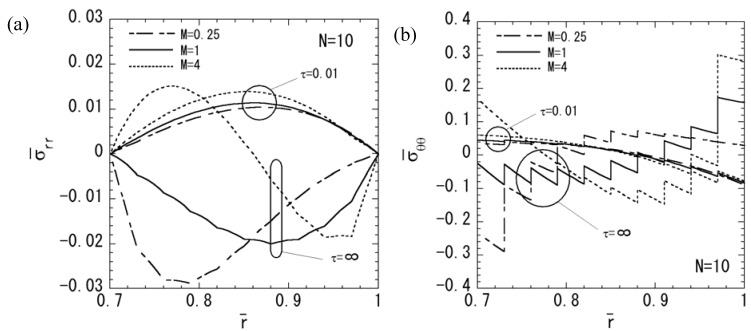
Variation of the thermal stresses (*N* = 10): (**a**) normal stress ${\bar{\sigma }}_{rr}$; (**b**) normal stress ${\bar{\sigma }}_{\theta \theta }$.

**Figure 7:**
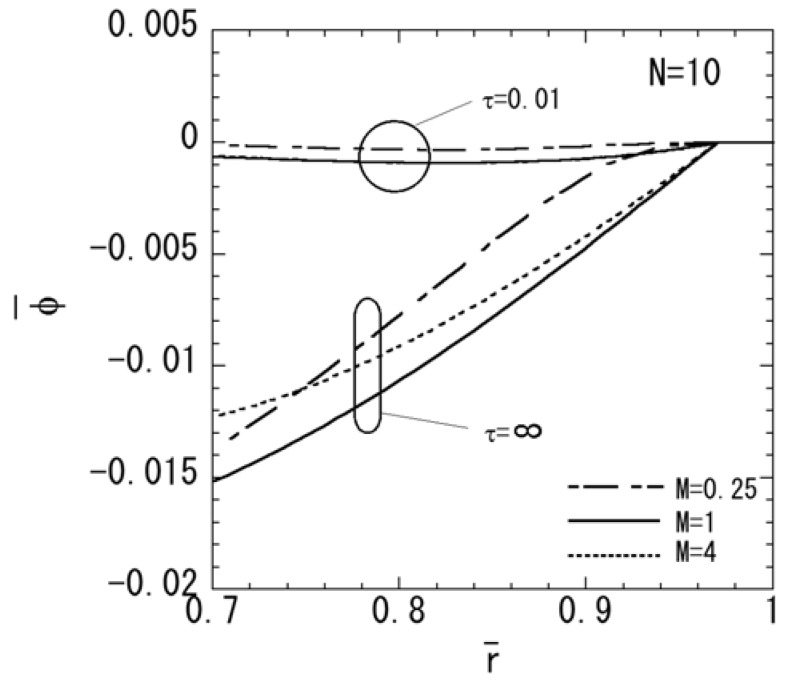
Variation of the electric potential (*N* = 10).

**Figure 8:**
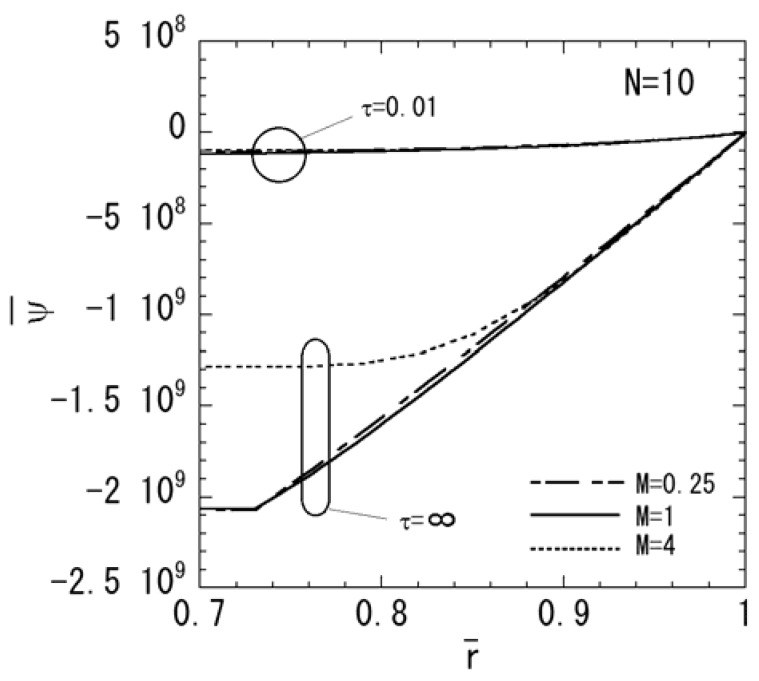
Variation of the magnetic potential (*N* = 10).

In order to assess the effect of the nonhomogeneous parameter *M* on the stresses, electric potential, and magnetic potential, the numerical results for *N* = 10 are shown in Figure 6, Figure 7 and Figure 8. *M* = 4 shows a piezoelectric material rich, and *M* = 0.25 shows a magnetostrictive material rich. The variations of stresses ${\bar{\sigma }}_{rr}$ and ${\bar{\sigma }}_{\theta \theta }$ are shown in Figure 6a and Figure 6b, respectively. From Figure 6a, it is clear that the maximum compressive stress of ${\bar{\sigma }}_{rr}$ decreases when the parameter *M* increases in the steady state. From Figure 6b, it is clear that the maximum tensile stress of ${\bar{\sigma }}_{\theta \theta }$ decreases when the parameter *M* decreases in the steady state. The variations of electric potential and magnetic potential are shown in Figure 7 and Figure 8, respectively. From Figure 7 and Figure 8, the absolute value of the electric potential in the inner surface is maximum when the parameter *M* = 1 in the steady state, while that of the magnetic potential is maximum when the parameter *M* = 0.25 in the steady state.

**Figure 9:**
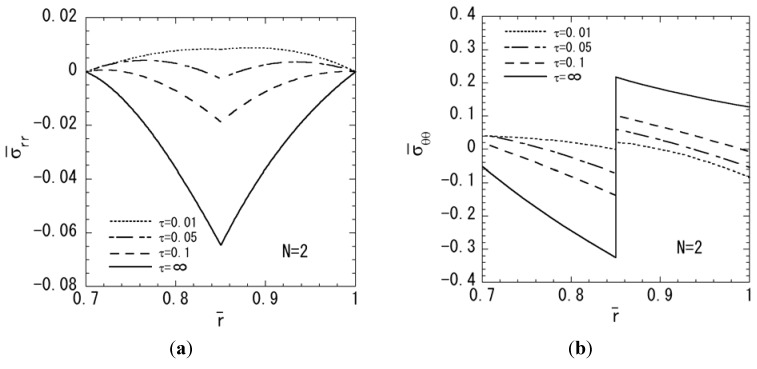

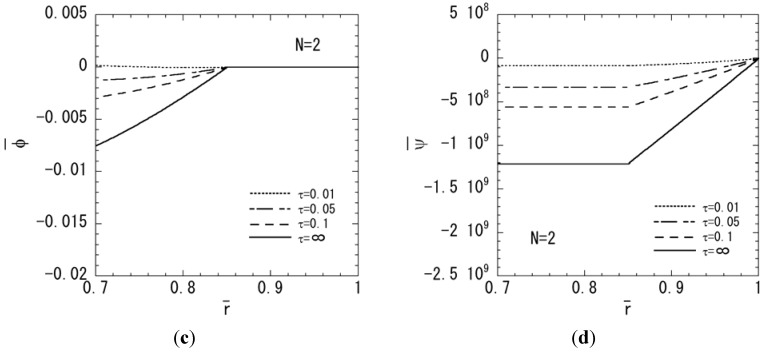
Numerical results for the two-layered hollow sphere (*N* = 2): (**a**) normal stress ${\bar{\sigma }}_{rr}$; (**b**) normal stress ${\bar{\sigma }}_{\theta \theta }$; (**c**) electric potential; (**d**) magnetic potential.

In order to assess the effect of relaxation of stress values in functionally graded magneto-electro-thermoelastic hollow sphere, the numerical results for the two-layered hollow sphere are shown in Figure 9. Figure 9a, Figure 9b, Figure 9c and Figure 9d show the variations of stresses ${\bar{\sigma }}_{rr}$, ${\bar{\sigma }}_{\theta \theta }$, electric potential and magnetic potential, respectively. From Figure 3 and Figure 9, the effect of relaxation of stress distributions for the functionally graded hollow sphere can be clearly seen compared with the two-layered hollow sphere. From Figure 4, Figure 5 and Figure 9, it is clear that the maximum absolute values of the electric potential and magnetic potential for functionally graded hollow sphere are grater than those for the two-layered hollow sphere.

## 4. Conclusions 

In this study, we analyzed the transient thermal stress problem for the functionally graded magneto-electro-thermoelastic hollow sphere due to uniform surface heating using a laminated composite mode by solving the governing equations of the displacement, electric potential and magnetic potential. As an illustration, we carried out numerical calculations for a functionally graded hollow sphere composed of piezoelectric BaTiO_3_ and magnetostrictive CoFe_2_O_4_, and examined the behaviors in the transient state for temperature change, displacement, stress, electric potential and magnetic potential distributions. We investigated the effects of the nonhomogeneity of material on the stresses, electric potential, and magnetic potential. Furthermore, the effect of relaxation of stress values in functionally graded magneto-electro-thermoelastic hollow sphere was investigated. We conclude that we can evaluate not only the thermoelastic response of the functionally graded magneto-electro-thermoelastic hollow sphere, but also the electric and magnetic fields of functionally graded magneto-electro-thermoelastic hollow sphere quantitatively in a transient state.
